# 

**Published:** 1856-02

**Authors:** 


EDITED BY

N. S. DAVIS, M.D.,

PROFESSOR OF PRINCIPLES AND PRACTICE OF MEDICINE AND CLINICAL MEDICINE IN RUSH MEDICAL COLLEGE; ONE OF THE PHYSICIANS TO THE MERCY HOSPITAL; FELLOW OF THE COLLEGE OF PHYSICIANS AND SURGEONS, NEW YORK; MEMBER OF THE MEDICAL SOCIETY OF THE STATE OF NEW YORK, AND OF THE STATE OF ILLINOIS; MEMBER OF THE AMERICAN MEDICAL ASSOCIATION, &C. &C.

AND

H. A. JOHNSON, A.M., M.D.,

PROFESSOR OF MATERIA MEDICA AND MEDICAL JURISPRUDENCE IN RUSH MEDICAL COLLEGE; ONE OF THE SURGEONS OF MERCY HOSPITAL; MEMBER OF THE AMERICAN MEDICAL ASSOCIATION; MEMBER OF THE ILLINOIS STATE MEDICAL SOCIETY, &C. &C.

FEBRUARY, 1856.

CHICAGO:

ROBERT FERGUS, PRINTER,

189 LAKE STREET.

VOL. XIII. WHOLE SERIES. No. 2.

